# Emergent Antidepressant Discontinuation Syndrome Misdiagnosed as Delirium in the ICU

**DOI:** 10.1155/2019/3925438

**Published:** 2019-08-04

**Authors:** Peter White, Carl L. Faingold

**Affiliations:** ^1^Division of Pulmonary & Critical Care Medicine, Department of Internal Medicine, Southern Illinois University School of Medicine, Springfield, IL, USA; ^2^Department of Pharmacology, Southern Illinois University School of Medicine, Springfield, IL, USA

## Abstract

We present a case of serious antidepressant discontinuation syndrome (ADDS) in a 72- year-old woman in the intensive care unit (ICU). Although this syndrome may be mild under ambulatory conditions, ICU patients can experience serious neurocognitive symptoms that are difficult to differentiate from delirium. We report delayed recognition of the ADDS in a patient in the ICU who was initially diagnosed with severe hyperactive delirium. Subsequent to hiatal hernia surgery, the patient was admitted to the ICU for aspiration and was intubated. Due to increasing agitation the patient received high doses of dexmedetomidine, fentanyl, midazolam, and propofol. The patient was treated with high doses of a serotonin norepinephrine reuptake inhibitor (SNRI) antidepressant, duloxetine, for 2 years. However, the antidepressant was not effectively administered postsurgically due to gastroparesis. The signs and symptoms of ADDS can occur cryptogenically if they are partially masked by sedating agents. Due to concern for the discontinuation syndrome and the inability to administer duloxetine via a nasogastric tube, another SNRI, venlafaxine, was administered. Venlafaxine administration allowed unexpectedly prompt dose reduction and then total discontinuation of all sedating agents, allowing liberation from mechanical ventilation and ICU discharge. This case illustrates the importance of avoiding antidepressant discontinuation in the ICU.

## 1. Introduction

Antidepressant drugs are one of the most common types of prescribed medications in patients prior to admission to the intensive care unit (ICU) [1]. To achieve effective treatment of depression this requires prolonged treatment with these agents.

Abrupt discontinuation of antidepressant drugs after chronic administration is known to lead to a discontinuation syndrome [2–6]. This has been observed with selective serotonin and norepinephrine reuptake inhibitors (SNRIs), as well as tricyclic antidepressant and selective serotonin reuptake inhibitors [4–6]. This discontinuation syndrome can become highly problematic if the patient is hospitalized for an extended period of time, especially in the ICU for an unrelated surgical or medical illness, and does not receive the antidepressant. Such patients can undergo the ADDS cryptogenically, because the syndrome can be partially masked by concurrent drugs such as opiate analgesics, benzodiazepines, or other sedating agents. Such an antidepressant discontinuation syndrome can even be mistaken for hyperactive delirium, which is a common but underrecognized diagnosis in ICU patients.

Delirium in mechanically ventilated patients in the ICU can often go undetected, because the changes in the patient's mental status may be attributed to the illness that caused the original hospitalization or from psychiatric causes [7]. Consistent identification of delirium requires the use of a reliable screening tool, such as the Confusion Assessment Method for the ICU (CAM-ICU) [8]. Risk factors for delirium include older age, hypertension, restraints, and sedating medications, all of which occurred in the present case. In addition, abrupt discontinuation of chronically administered psychoactive medications can be associated with delirium in ICU patients.

Delirium associated with discontinuation of a psychoactive drug is well recognized in the case of chronic alcohol, benzodiazepine, or opiate exposure [9]. However, ADDS is less well recognized, especially in the context of the ICU. It is common practice in the ICU to discontinue “unnecessary” medications in patients with critical illness to avoid drug-drug interactions with the medications needed during the period of critical illness. Despite the fact that abrupt discontinuation of antidepressants will precipitate ADDS, this issue has received little attention by critical care practitioners. The following is a case report of extreme agitation temporally associated with antidepressant discontinuation in the ICU and the subsequent rapid resolution of the agitation when an antidepressant, acting by the same mechanism of action, was administered. The patient's symptoms were not typical for the ADDS seen under ambulatory conditions, but they are a dramatic and notable example of how serious this syndrome can become in the ICU. This case illustrates the need for critical care physicians to be familiar with the ADDS and to consider the diagnosis in agitated ICU patients who have recently undergone antidepressant discontinuation.

## 2. Case Presentation

The patient is a 72-year-old female, never cigarette smoker, with an extensive medical history, including treatment for COPD/asthma, obstructive sleep apnea, coronary artery disease (S/P coronary artery bypass surgery), hypertension, hypothyroidism, and gastroesophageal reflux disease. She also had a large hiatal hernia (> 50% of the stomach was in the chest), which was the initial cause of her hospitalization. She had been treated for depression with duloxetine, a SNRI (extended release), 60 mg (0.6 mg/kg) twice daily for two years as an outpatient. She was subject to progressive exertional breathlessness, which was attributed to the combination of hiatal hernia, COPD/asthma, and obesity. She was hospitalized, and a laparoscopic hiatal hernia repair (Toupet antireflux fundoplication and gastropexy) was performed.

Postoperative day (POD) 0 to 12: the patient returned to the operating room on POD 1 due to recurrence of the hiatal hernia and the development of extensive pneumomediastinum. A torn suture and associated small tear in the stomach were identified. The fundoplication was taken down and redone.

The CAM-ICU was negative on POD 0 to 5 and 7 to 8, not recorded on POD 10 to 12, and positive on POD 6. The patient had an endotracheal tube for positive pressure mechanical ventilation removed on POD 2, reinserted on POD 5, removed on POD 6,and reinserted on POD 12. She was reported to have received duloxetine (120 mg, orally) on POD 0 and 1, 8, 9, 10, 11, and 12. However, she was subject to frequent bouts of emesis due to gastroparesis, and it is unclear how much of the drug was absorbed. On POD 12 emesis resulted in aspiration, which caused respiratory failure, reintubation, and emergent readmission to the ICU. She had been sedated with dexmedetomidine on POD 0, 1, and 2, propofol on POD 5, and both medications and restraints were required on POD 12 for stabilization of the patient in the ICU. Propofol was initially discontinued on POD 13. During this admission to the ICU she was treated for hospital acquired pneumonia with vancomycin and piperacillin-tazobactam.

The CAM-ICU was positive on POD 13 to 25 except for POD 16, 23, and 24 when it was not recorded. She required sedation (various combinations of dexmedetomidine, fentanyl, and midazolam; see Table 1 for details) for agitation from POD 13 to 25. On POD 20 these sedating drugs were insufficient, and propofol was needed to prevent unplanned extubation. During POD 13 to 24 the patient could not tolerate pressure support spontaneous breathing trials. Her appearance was described by the respiratory therapists as “frantic” or “panicked” when they attempted to determine the rapid shallow breathing index. Duloxetine was not given as POD 13. However, due to concern for SNRI ADDS, on POD 20 the contents of the duloxetine capsule were crushed for administration, but the duloxetine pellets would not pass through the nasogastric tube. However, another SNRI, venlafaxine (crushed 75 mg tablet immediate release), which was able to pass via the nasogastric tube, was administered three times daily. As the daily dose of venlafaxine was increased, the doses of the sedating drugs required to prevent unplanned extubation were reduced; until by POD 25 these agents were completely discontinued, and the patient was liberated from mechanical ventilation.

On POD 28 to 36 the CAM-ICU was negative. The patient was not agitated or confused. She remained on venlafaxine and received lorazepam (1 mg orally) on POD 33, 35, and 36 for sleep. Otherwise she did not require medication for pain, agitation, or delirium, and she was transferred out of the ICU.

## 3. Discussion

This patient who was undergoing unintended discontinuation of an antidepressant was initially diagnosed in the ICU as suffering from agitated delirium. Delirium typically consists of altered cognition, inattention, or inability to maintain focus, fluctuating course throughout the day, and rapid onset (usually hours to days) [10].

Delirium is often associated with acute physiologic stress, such as a systemic inflammatory or infectious illness, profound metabolic disturbance, or withdrawal from a drug or alcohol [11]. It is estimated that up to 80% of mechanically ventilated patients and up to 50% of nonmechanically ventilated patients in the ICU suffer from delirium, which is associated with significant morbidity [12]. In mechanically ventilated patients unplanned extubation is 5 times more likely, reintubation following a planned extubation is 3 times more likely, and length of stay in the ICU and hospital are longer due to delirium [13]. In addition, the duration of mechanical ventilation is longer, and the severity of illness is increased due to delirium. Mortality increases 10% for every additional day of delirium in the ICU [14]. Long-term sequelae of delirium include functional disability, cognitive impairment, need for institutional care, onset of dementia after discharge, and increased incidence of death at 6 and 12 months [12]. In the present case, mechanical ventilation was prolonged, and it was difficult to keep the patient sedated despite relatively high doses of midazolam, fentanyl, and dexmedetomidine, and eventually propofol was required until after an SNRI was reintroduced. The patient died less than 6 months after this hospitalization.

The ADDS that occurs in the ICU can be difficult to differentiate from delirium, especially in a nonverbal mechanically ventilated patient, in part because neither syndrome has a specific diagnostic test. Although the CAM-ICU screening tool has proven useful to diagnose confusion [2, 8, 15], confusion is seen in both delirium and ADDS. The patients' drug history of antidepressant usage is a critical indicator in suspected ADDS. Once ADDS is suspected it can be most effectively managed by reintroduction of the specific drug that the patient had been taking and evaluating if the symptoms diminish in intensity. However, as in this case, when this is not feasible, the next best alternative is administration of a drug that acts by the same mechanism [16]. Thus, the available formulations of duloxetine, the SNRI in this case, which has a high incidence of ADDS [17], could not be reliably administered via a nasogastric tube, but another SNRI, venlafaxine, which is available in a crushable dosage form, was effective in resolving the agitated and confused state relatively rapidly. During several days of venlafaxine treatment, the delirium lifted completely, allowing the doses of sedatives to be progressively reduced and then eliminated, allowing successful extubation.

The ADDS develops because the changes in the action of brain biogenic amines induced by these agents are reversed [6] following the abrupt discontinuation of the administration of antidepressants of many different types, including SNRIs, in part because these agents require long-term administration to be effective [1, 18–20]. ADDS is defined by the following clinical criteria: (1) abrupt discontinuation or dose reduction of an antidepressant after use for more than 4 weeks, (2) onset of 2 or more typical symptoms (flu-like symptoms such as lassitude, malaise, headache, myalgia, and diarrhea; insomnia; nausea; imbalance, gait instability, dizziness, lightheadedness, and vertigo;** s**ensory disturbances such as paresthesia, electric shock sensations with head movement, “Brain Zaps,” blurred vision, and other visual disturbances; and** h**yperarousal symptoms such as anxiety or agitation; FINISH mnemonic) [3] within 1-7 days after a change or discontinuation in the medication dosing that results in significant social or occupational distress, (3) resolution of the symptoms with reinitiating the original antidepressant or a similar medication, and (4) the patient's symptoms which are not better explained by recurrence of depression, another illness, or change in another medication [18–20]. The symptoms are not due to drug addiction. Antidepressants are not habit forming, and patients do not demonstrate drug seeking behavior. It is estimated that 25% to 30% of patients who abruptly stop or significantly decrease the dose of an antidepressant will experience discontinuation symptoms [2] (Table 2). It is commonly held that these symptoms are mild and short-lived (resolving in 2 to 3 weeks) and that on average symptoms are more common with antidepressants that have short half-lives. However, the spectrum, intensity, and/or duration of an individual's discontinuation symptoms are variable and are patient- not drug-specific. If these symptoms occur under critical care conditions due to unrelated causes, they can potentially result in severe agitation and neurocognitive dysfunction that is intense and debilitating and can be mistaken for ischemic stroke [2] or agitated delirium, as was the case for this patient.

This patient satisfies all the diagnostic criteria for a SNRI ADDS. She had taken duloxetine for 2 years, and it was abruptly discontinued when she was NPO due to mechanical ventilation. She experienced profound agitation and emotional distress within 72 hours of not receiving duloxetine, requiring sedation with a combination of dexmedetomidine, midazolam, and fentanyl in the highest doses normally employed in the ICU, and finally propofol was required to prevent unplanned extubation. Her symptoms were mistaken for agitated delirium. Five days after restarting a substitute SNRI medication (venlafaxine) her agitation completely resolved, and she was liberated from mechanical ventilation and transferred out of the ICU in spite of developing fungemia due to* Candida albicans *line sepsis.

Numerous studies indicate that the abrupt discontinuation of chronic psychoactive medications, such as benzodiazepines or opioids, is associated with the development of delirium in ICU patients [7]. Critical Care physicians routinely discontinue other medications that are deemed not acutely necessary in patients with critical illness. It is known that abruptly discontinuing antidepressants may precipitate discontinuation symptoms in approximately one third of patients [2, 21]. However, this issue has received little attention in the ICU and deserves greater vigilance in critical care patients who have been taking antidepressants chronically, as seen in the present case.

There are weaknesses to our conclusion that this patient suffered from the ADDS. She had multiple medical comorbidities, had recently undergone 2 major surgeries, was critically ill, experienced acute respiratory failure requiring mechanical ventilation, and received high doses of sedative agents. There were issues with gastric motility and, while she would take an oral medication, she often vomited so it was unclear to what extent the drug was absorbed. Further, she was CAM-ICU positive and was treated with olanzapine. She had major risk factors (age, immobility, and sedation with benzodiazepines) for delirium. However, there is no convincing evidence that the duration of delirium in the ICU is shortened by treatment with atypical antipsychotic agents such as olanzapine [9]. The time course of her cognitive improvement after SNRI readministration was unexpectedly fast for delirium in the ICU [9, 11].

In conclusion we believe that this patient's agitation was primarily due to ADDS, not delirium, because its severity required very aggressive treatment with high doses of sedating drugs and the relatively rapid improvement after readministration of a related antidepressant. Critical Care physicians routinely discontinue what they perceive as “unnecessary” medicines, including antidepressant and antipsychotic medications. The potential consequence of abruptly discontinuing antidepressant medications and the need for promptly resuming treatment are underappreciated in critically ill patients.

## Figures and Tables

**Table 1:** Richmond Agitation Sedation Scale (RASS), Behavioral Pain Scale (BPS) Confusion Assessment Method for the ICU (CAM-ICU), and selected medications for postoperative days 10 – 27.

	POD 10	POD 11	POD 12	POD 13	POD 14	POD 15	POD 16	POD 17	POD 18	POD 19	POD 20	POD 21	POD 22	POD 23	POD 24	POD 25	POD 26	POD 27
RASS, max./min.	0/0	1/1	-1/-2	-1/-2	1/-2	1/-2	3/-3	-1/-1	1/-3	2/-3	3/-4	2/-2	1/1	-1/-1	-1/-1	0/0	0/0	0/-1
BPS, max.	N/A	N/A		0	1	6	8	8	0	4	9	8	2	2	1	1	N/A	N/A
CAM-ICU	N/A	N/A	No	Yes	Yes	Yes	N/A	Yes	Yes	N/A	Yes	Yes	Yes	N/A	N/A	Yes	No	No
Fentanyl gtt max/min (IVP)	(700)	-* *-* *-	-* *-* *-	250/200	250/150 (25)	200/200	250/150	250/200	250/200	250/250	250/200	200/50	200/50	90/50	70/0	-* *-* *-	-* *-* *-	-* *-* *-
Midazolam max/min (IVP)	-* *-* *-	-* *-* *-	-* *-* *-	4/3	4/2	6/1	8/0	8/4	8/4 (5)	8/3	8/1	4/0	5/0	-* *-* *-	-* *-* *-	-* *-* *-	-* *-* *-	-* *-* *-
Dexme gtt max/min	-* *-* *-	-* *-* *-	1/0	1.2/0	-* *-* *-	-* *-* *-	1.2/0	0.80.2	1/0.2	1.2/0.6	1.2/0.4	0.6/0	0.6/0.6	-* *-* *-	-* *-* *-	-* *-* *-	-* *-* *-	-* *-* *-
Propofol max/min	-* *-* *-	-* *-* *-	15/0	-* *-* *-	-* *-* *-	-* *-* *-	-* *-* *-	-* *-* *-	-* *-* *-	-* *-* *-	15/0	50/15	50/30	40/25	40/20	30/0	-* *-* *-	-* *-* *-
Duloxetine	120	120	120	0	0	0	0	0	0	0	120 (crushed)	0	120 (crushed)	0	0	0	0	0
Venlafaxine	0	0	0	0	0	0	0	0	0	0	75	75	150	150	225	225	150	300
Olanzapine	0	0	0	0	0	0	0	0	7.5	15	7.5	15	15	15	15	15	7.5	0

Abbreviations

RASS: Richmond Agitation Sedation Scale

BPS: Behavioral Pain Scale

CAM-ICU: Confusion Assessment Method for the ICU

dexme: dexmedetomidine

max.: the maximum IV dose (mcg for fentanyl and mg for midazolam)/infusion rate (mcg /hr for fentanyl, mcg/kg/hr for dexmedetomidine, and mcg/kg/min for propofol)

min.: the minimum IV dose/infusion rate (dosing units and concentrations the same as for maximum).

IVP: IV push medication

gtt: IV infusion.

Duloxetine, venlafaxine, and olanzapine daily dose is in mg.

**Table 2:** Signs and symptoms of antidepressant discontinuation syndrome.

	SSRI	Atypical antidepressant	Tricyclic antidepressant	MAOI
*General*				
Flu-like symptoms	+	+	+	–
Headache	+	+	+	+
Lethargy	+	+	+	-
*Gastrointestinal*				
Abdominal cramping	+	–	+	–
Abdominal pain	+	–	+	–
Appetite disturbance	+	+	+	–
Diarrhea	+		+	–
Nausea/vomiting	+	+	+	–
*Sleep*				
Insomnia	+	+	+	+
Nightmares	+	+	+	+
*Balance*				
Ataxia	+	–	+	–
Dizziness	+	+	+	–
Lightheadedness	+	–	+	–
Vertigo	+	+	+	–
*Sensory*				
Blurred vision	+	–	–	–
“Electric shock” sensations	+	+	–	–
Numbness	+	–	–	–
Paresthesia	+	+	–	–
*Movement*				
Akathisia	+	+	+	–
Myoclonic jerks	–	–	–	+
Parkinsonism	+	–	+	–
Tremor	+	–	+	–
*Affective*				
Aggression/irritability	+	–	–	+
Agitation	+	–	+	+
Anxiety	+	+	+	–
Low mood	+	+	+	+
*Psychosis*				
Catatonia	–	–	–	+
Delirium	–	–	–	+
Delusions	–	–	–	+
Hallucinations	–	–	–	+

Note: symptom categories are listed by rate of incidence.

SSRI = selective serotonin reuptake inhibitor; MAOI = monoamine oxidase inhibitor: + = occur in withdrawal from this medication; – = do not occur in withdrawal from this medication. Table 2 is reproduced from Warner et al., 2006, under the Creative Commons Attribution License/public domain.

## Data Availability

Relevant data and supporting material are available from the corresponding author on reasonable request.

## Abbreviations

BPS: Behavioral Pain Scale
CAM-ICU: Confusion Assessment Method for the ICU
COPD: Chronic obstructive pulmonary disease
dexme: Dexmedetomidine
gtt: IV infusion
ICU: Intensive care unit
IVP: IV push medication
POD: Postoperative day
RASS: Richmond Agitation Sedation Scale
SNRI: Serotonin norepinephrine reuptake inhibitor.
